# Meta-populational demes constitute a reservoir for large MHC allele diversity in wild house mice (*Mus musculus*)

**DOI:** 10.1186/s12983-018-0266-9

**Published:** 2018-04-20

**Authors:** Miriam Linnenbrink, Meike Teschke, Inka Montero, Marie Vallier, Diethard Tautz

**Affiliations:** 1 Present address: Deutsche Forschungsgemeinschaft, 53170 Bonn, Germany; 2 Present address: Medical Faculty, Eberhard Karls Universität Tübingen, Tübingen, Germany; 3 Max-Planck Institute for Evolutionary Biology, August-Thienemannstrasse 2, 24306 Plön, Germany

**Keywords:** Major histocompatibility complex, House mouse, Natural populations, Recombination, Reservoir model

## Abstract

**Background:**

The MHC class I and II loci mediate the adaptive immune response and belong to the most polymorphic loci in vertebrate genomes. In fact, the number of different alleles in a given species is often so large that it remains a challenge to provide an evolutionary model that can fully account for this.

**Results:**

We provide here a general survey of MHC allele numbers in house mouse populations and two sub-species (*M. m. domesticus* and *M. m. musculus*) for H2 class I D and K, as well as class II A and E loci. Between 50 and 90% of the detected different sequences constitute new alleles, confirming that the discovery of new alleles is indeed far from complete. House mice live in separate demes with small effective population sizes, factors that were proposed to reduce, rather than enhance the possibility for the maintenance of many different alleles. To specifically investigate the occurrence of alleles within demes, we focused on the class II H2-Aa and H2-Eb exon 2 alleles in nine demes of *M. m. domesticus* from two different geographic regions. We find on the one hand a group of alleles that occur in different sampling regions and three quarters of these are also found in both sub-species. On the other hand, the larger group of different alleles (56%) occurs only in one of the regions and most of these (89%) only in single demes. We show that most of these region-specific alleles have apparently arisen through recombination and/or partial gene conversion from already existing alleles.

**Conclusions:**

Demes can act as sources of alleles that outnumber the set of alleles that are shared across the species range. These findings support the reservoir model proposed for human MHC diversity, which states that large pools of rare MHC allele variants are continuously generated by neutral mutational mechanisms. Given that these can become important in the defense against newly emerging pathogens, the reservoir model complements the selection based models for MHC diversity and explains why the exceptional diversity exists.

**Electronic supplementary material:**

The online version of this article (10.1186/s12983-018-0266-9) contains supplementary material, which is available to authorized users.

## Background

Genes encoded in the Major Histocompatibility Complex (MHC) of vertebrates play key roles for the adaptive immune system. By presenting antigens of processed peptides from pathogens to T lymphocytes, proteins encoded in the MHC initiate a specific direct immune response. Among MHC Class I and II genes one can find the most polymorphic loci known in the vertebrate genome [1, 2]. Despite decades of intense research, the evolutionary mechanisms of the origin and the maintenance of a large number of alleles in a given species are still much debated [3–11]. The two main adaptive factors that contribute to the maintenance of large MHC allele numbers are thought to be parasite-mediated selection and sexual selection [9], but theoretical studies have shown that these could account only for dozens to hundreds of alleles at a given locus [6, 7], but not the thousands that are nowadays found in deep surveys [12–14].

The most detailed data for MHC allele numbers are available for humans, since they are routinely analyzed in large cohorts to find matching donors for hematopoietic stem cell transplants. By surveying the human allele numbers, Klitz et al. [12] have suggested that the large numbers are due to recombination mechanisms that continuously generate new variants at low frequencies, but which are important to serve as a reservoir of alleles to fight new pathogens when they arise. The most recent re-analysis of human alleles has confirmed that millions of alleles exist in human populations, composed of a core set of ancient alleles, a much larger group of recombinant alleles (tens of thousands) and a long tail of extremely rare alleles that represent de novo point mutations in single individuals [13, 14]. Robinson et al. [14] point out in their discussion that “... understanding their genetics and biology in any species requires extensive study of populations. For reasons of cost and logistics this has been rarely, if ever, achieved. Many population studies have recruited only small numbers of individuals (therefore, likely missing rare alleles)...”. Our study here provides such an extended allelic survey in natural populations of the house mouse (*Mus musculus*), including a dedicated sampling scheme to explore MHC alleles within a metapopulational context.

The MHC complex in mice is located on chromosome 17 and is called H2 (or H-2) [15]. In a classic paper on heterozygosity of H2 loci in wild mice, Duncan et al. [16] found a close to 100% heterozygosity for class I loci and estimated the existence of at least 100 segregating alleles. However, this high heterozygosity stood in contrast to the known structure of inbreeding within demes. They wrote that “The intrademic inbreeding should lower the heterozygosity at the H-2 loci...” and “The deme structure of the mouse population and the high degree of heterozygosity need to be reconciled” [16]. As a possible solution, they proposed that heterozygote advantage should offset the effect of inbreeding.

While a large number of segregating alleles in mice was later confirmed through various approaches [17–22] the analysis of allele distribution in demes was not revisited. Further, modeling of the heterozygote advantage has shown that it cannot explain the large number of alleles at H2 loci on its own [6, 7]. Rare-allele advantage and fluctuating selection may further contribute to the maintenance of larger allele numbers, but these factors are difficult to disentangle [9].

The phylogenetic and phylogeographic history of house mice has been well studied [23–25]. They have their origin in Iran or India and they have split into several subspecies within the past half million years. The focus in the present study are the Western (*M. m. domesticus*) and the Eastern (*M. m. musculus*) subspecies that have colonized their current territories only a few thousand years ago, as commensals with the spread of humans and establishment of agriculture. *M. m. domesticus* is thought to have arrived in Western Europe about 3000 years ago, originating from a distinct population in Western Iran [25–27]. The colonization of Eastern Europe and Northern Asia by *M. m. musculus* may have occurred a few thousand years earlier, possibly starting from a source population in Eastern Iran. The *M. m. domesticus* spread across Western Europe has lead to a pattern of highly differentiated subpopulations [27–30]. In spite of their spatial differentiation, some gene flow occurs between populations and subspecies, apparently due to adaptive introgression of advantageous haplotypes across large distances [31, 32] as well as across the hybrid zone in Central Europe [33].

Here we use two sampling schemes to study MHC allele numbers in house mice. One is an overall survey across populations of the two sub-species, *M. m. domesticus* and *M. m. musculus*, the other is a specific survey of class II alleles within demes of two geographically distinct populations of *M. m. domesticus*. The overall survey yields many new alleles that were not previously described, but confirms also the sharing of a subset of alleles between the sub-species. Surprisingly, the survey of the demes revealed even higher allele numbers, with many alleles occurring only within single regions or demes and relatively little sharing between demes located next to each other. Analysis of these alleles suggest that they have arisen mostly through recombination mechanisms between existing alleles. Our data show that demes can serve as reservoirs of new alleles and support the inferences derived from human MHC allele surveys that at least thousands of rare alleles may exist that can become relevant for immune defense when new pathogens arise.

## Methods

### Ethics and permissions

This work did not involve research on animals that would require permission by an ethics committee. The trapping and keeping of animals was done under the permission of the authorities (permit from Veterinäramt Kreis Plön: 1401–144/PLÖ-004697), according to §11 of the German animal welfare law (Tierschutzgesetz). Cervical dislocation was used when mice had to be sacrificed. Mice were either caught in snap traps, or live traps (“Mäusewippfalle” No. 3451002, Firma Ehlert & Partner, 53,859 Niederkassel, Germany) and live mice were kept according to the FELASA guidelines as described in Harr et al. 2016 [27].

### Sampling

**Fig. 1:**
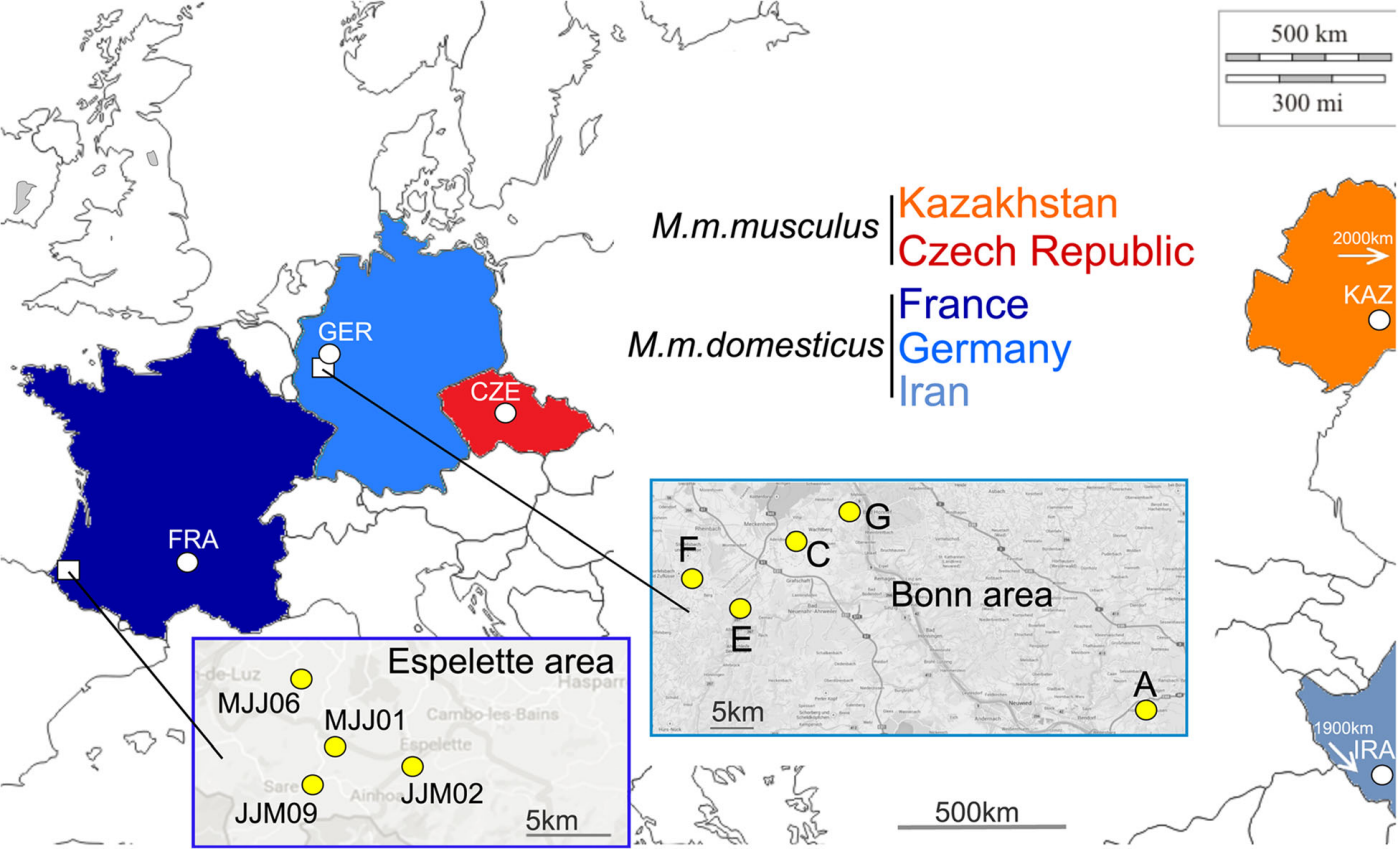
Sampling locations for sample sets 1 and 2. Sample set 1 consists of population samples from five countries, three of the subspecies *M. m. domesticus* (blue shades) and two of the sub-species *M. m. musculus* (red shades). The respective sampling sites in each country are marked by white dots. Sample set 2 includes the demes near Bonn (Germany - overlaps with the Cologne/Bonn region samples of sample set 1) and Espelette (France) shown as insets with their respective sampling sites as yellow dots

Sample set 1 was used to generate a general overview of allele diversity, based on a 454 sequencing approach. We focused on two subspecies of the house mouse (*M. m. domesticus* and *M. m. musculus* (Fig. 1). *M. m. domesticus* is represented by individuals from Iran (IRA), from the Cologne/Bonn region in Germany (GER) and from the Massif Central region (FRA) in France. The population from Iran is considered to represent the ancestral source population for *M. m. domesticus* in Western Europe [25]. *M. m. musculus* is represented by individuals from the Czech Republic (CZE) and Kazakhstan (KAZ), whereby the KAZ population is considered to be the more ancestral one. DNA samples for sample set 1 were obtained from tissue of wild mice, caught in 2004/2005 with a collection regime that aimed to sample the diversity across a region [28]. For the present study we used 12 individuals from GER, IRA, KAZ and CZE and 25 from the FRA population, in total 73 individuals.

Sample set 2 was used to assess the maintenance of allelic diversity in meta-populational demes. These samples were obtained from a region in Germany (Bonn) and a region in France (Espelette) (Fig. 1). The German region overlaps with the region of sample set 1, but the mice were caught in 2012. The French region was different from the one in sample set 1 and was sampled in 2013. Mice for sample set 2 were caught under a different sampling scheme than the mice for sample set 1. Instead of maximizing diversity by using only a single mouse from each location, we aimed to obtain multiple individuals from the same locality, i.e., farms in sample set 2, including the possibility to catch some offspring from the same family. 6–18 individuals per farm were caught within a few days each, which resulted in a sample set of 47 mice from Bonn and 46 from Espelette. MHC alleles for these samples were determined by two different procedures, namely direct sequencing of PCR fragments for each individual and Illumina sequencing of PCR fragments from the same individuals.

### Targeted loci

We focused on two MHC class I (H2-D and H2-K) and 4 MHC class II loci (H2-Aa, H2-Ab, H2-Ea and H2-Eb). We targeted the following exons: H2-D Exon 2 and 3, H2-K Exon 2, 3 and 4, H2-Aa Exon 2 and 3, H2-Ab Exon 2 and 3, H2-Ea Exon 2 and 3, H2- Eb Exon 2 and 3 (Additional file 1: File S1 provides an overview). Exons 2 and 3 of MHC class I loci and exon 2 of class II loci code for the antigen peptide binding groove. PCR primers were designed manually by searching exon flanking intronic regions for suitable sequences in the mouse reference sequence [34]. For each exon, up to four primer pair combinations were used for each animal that spanned the complete exons of each of the above mentioned loci to be able to confirm alleles in different PCR fragments. A list of all primers and PCR conditions used in this study is provided in Additional file 2: Table S1.

### 454 Library preparation strategy

MHC allele detection through parallel sequencing, such as 454 sequencing, is superior to more indirect methods such as techniques based on physical separation of alleles, e.g., single-stranded conformational polymorphism (SSCP), denaturing gel gradient electrophoresis (DGGE), or reference strand mediated conformational polymorphism (RSCA) [35]. But there are also drawbacks, such as errors introduced by the sequencing technique [35, 36], as well as PCR errors caused by the high levels of heterozygosity. PCR reactions to amplify MHC loci are effectively carried out with multi-allelic templates, which frequently cause problems, mainly through the formation of chimeras through incompletely extended primers, which act as primers in the subsequent PCR cycles [37, 38]. Especially in the later cycles of the PCR, this chimera formation increases when the concentration of the incompletely extended primers is high enough to compete with the original primers for annealing. Thus, artificially recombined alleles are generated. This problem increases when templates of different individuals are amplified in the same PCR reaction, especially for the extremely polymorphic MHC Class II alleles. To minimize the problems caused by possible PCR artifacts, we reduced the number of PCR cycles to 28 and used independent amplifications. The first step of library preparation was the amplification of MHC exon fragments in individual PCRs. Each fragment was separately amplified from each individual in four independent reactions using the different primer combinations described above. After determining the concentrations of each PCR per locus via band intensity after gel electrophoresis and measuring the concentration on a Qubit fluorometer using the Quant-iT BR assay (Invitrogen), all fragments per individual were normalized to the same concentration and pooled. Pools were then tagged by ligating multiplex identification tags (MIDs) of 10 bp to the fragments. The last step consisted of the ligation of amplicons with Y adapters and then 454 sequencing according to the standard protocol (Roche). A total of three pools were generated and each was run on one lane. Pool 1 encompassed 25 individuals of the FRA population and yielded 89,782 reads, pool 2 encompassed 12 individuals each of the CZE and KAZ populations and yielded 91,498 reads, pool 3 encompassed 12 individuals each of the GER and IRA populations and yielded 92,006 reads.

### Raw read processing

After quality filtering of reads by applying the criteria of average quality score for bases > 25 and read length > 200 bp, without any “Ns”, the remaining reads were edited with BioEdit. The MID-tags were cut off the sequences. Forward and reverse sequences were determined, primers and the intronic regions close to the exon were trimmed. Read statistics are provided in Additional file 3: Table S2. In the last step of data processing, all reads per locus were aligned to reference sequences obtained from database surveys using ClustalW [39]. Typical sequencing errors as described by Babik et al. [35] were manually edited in all full length reads (Additional file 1: File S1 for detailed description and examples). After raw read processing, alleles were identified as being reliable if the same sequences were found in at least two independent amplifications with different primers or in two different individuals. As a final step, the sequences were checked back against the un-edited raw reads. If at least two raw reads confirmed the sequence, it was accepted. All reliable alleles per locus are listed in Additional file 4: Table S3 (class I loci) and Additional file 5: Table S4 (class II loci).

### Sanger sequencing of exons

The allele assignment for the sample set 2 individuals was done by direct sequencing of PCR fragments of H2-Aa and H2-Eb exon 2 from individuals through Sanger sequencing, which was previously shown to be efficient for determining the sequence of MHC alleles and which avoids also the problem of PCR recombination artifacts [40]. Primers and the PCR protocols are included in Additional file 2: Table S1. All individuals were sequenced separately and sequences were edited manually with Codon Code Aligner and aligned with ClustalW, included in the program MEGA 6 [41]. To provide raw sequence information for both loci two project files were generated in Codon Code Aligner. Heterozygous positions were identified based on double peaks in the sequence reads, both through automatic calling, as well as manual curation, since we noted that automatic calling does not detect all heterozygous positions. Haplotypic phase was determined using PHASE version 2.1 [40, 42, 43] and manually curated, based on known alleles. In some cases we used also the inequality of peak sizes in the overlapping sequences, due to unequal amplification of the alleles, as additional criterion for phasing.

### Illumina sequencing

To further confirm the alleles and the correct phasing of alleles of set 2, we used an Illumina sequencing approach which can provide faithful results [44]. We amplified DNA from all sample set 2 individuals with the primers included in Additional file 2: Table S1, including unique barcoding tags. The fragments were then run on an Illumina MiSeq, using the long read kit to obtain single sequences across the whole region. Only individuals with > 5000 reads were considered in the analysis. To detect whether one or two alleles were present, UPGMA trees of the aligned reads were constructed and checked whether one or two clusters were present. This allows to identify also cases where the two alleles were amplified very unequally. Consensus sequences were then generated across the reads of the clusters.

### Consolidation of sample set 2 reads

The direct and Illumina obtained haplotypes for each individual were compared to see whether they validate each other. Conflicts were resolved by re-inspecting the original direct reads. However, we found also cases where the two approaches did not validate each other, apparently due to poor amplification of one of the two alleles. We had a few cases where the direct sequencing showed clear peaks (i.e. no indication of a second allele), while the Illumina reads showed clearly two alleles and vice versa. In some cases we could verify the second allele inferred from the direct sequencing by directly searching it among the Illumina reads, i.e. it was in these cases only very poorly amplified. We have therefore recorded for each individual whether the respective alleles were supported by both approaches, or only one of them (the latter also partly due to failure to get a result for the respective individual from one of the approaches) (Additional file 6: Table S5 and Additional file 7: Table: S6). Individuals for which we had no Illumina verification and for which we could extract no known allele from the direct sequencing were not considered in the final analysis, since phase could not be determined. This implies that we are in fact somewhat underestimating the number of alleles in the demes. Further, because of the different precautions and checks that were applied, we can practically rule out the possibility that some alleles may be due to PCR recombination artifacts.

All reliable alleles were compared with those identified in sample set 1 and with previously recorded alleles in databases using alignments and UPGMA trees generated in Geneious (Geneious.com). To make them comparable, alleles were all trimmed to the same length (208 bp for H2-Aa and 231 bp for H2-Eb), which is shorter that the whole exon due to missing data in some individuals.

### Microsatellite genotyping

To infer relatedness among individuals of sample set 2, we chose 10 unlinked microsatellite markers derived from Thomas et al. [45]. These markers are Chr3_24R, Chr16_21R, CHr12_05R, Chr01_25R, Chr17_09R, Chr05_45R, Chr13_22R, Chr19_08R, Chr14_16R, Chr09_20R. Primer information is given in Additional file 2: Table S1. Forward primers were labeled with FAM or HEX and PCR was performed using 5 ng/μL DNA template together with the Multiplex PCR kit (QIAGEN). After processing PCR products with HiDi formamide and 500 ROX size standard, samples were run on an ABI 3730 sequencer (Applied Biosystems). Raw alleles were called using GeneMapper 4.0 (Applied Biosystems). The alleles are provided in Additional file 8: Table S7.

### Statistics

Observed and expected heterozygosities for microsatellites were calculated using MSA version 4.05 [46] and for MHC alleles using GENODIVE [47]. Average relatedness was determined with the program COANCESTRY [48]. To investigate the structure between and within the farms we used the software STRUCTURE [49] for the microsatellite data. The parameters used were 500,000 burn-in period and 1,000,000 Markov Chain Monte Carlo (MCMC) simulations with 10 iterations per number of clusters (K) for K equals 2–15. To determine the most likely number of populations that describe the existing amount of genetic structure, we used Structure Harvester [50] and applied the criterion of Evanno et al. [51]. Calculations of Hardy-Weinberg equilibrium tests between demes were performed in Arlequin [52]. Rarefaction analysis was done with the R-package “vegan” using the function “rarefy” with 10,000 bootstrap. dN/dS ratios were calculated in MEGA 6.0 [41], distances are based on the Kimura 2 Parameter model. The minimum number of recombination events and determination of sites between which recombination is inferred was investigated by using the software DNASp [53] and the genetic algorithm for recombination detection (GARD) as implemented on the webserver DATAMONKEY [54]. The software GENECONV (http://www.math.wustl.edu/~sawyer) [55] was used to identify possible past gene conversion events. Significant gene conversion tracks were detected by permutation test (1,000,000×) and *p*-values were corrected for multiple comparisons.

### D-loop sequencing and analysis

A fragment of ~ 980 bp of the mitochondrial D-loop was sequenced. Primers used were taken from Prager et al. [56] (Additional file 2: Table S1). Sequences were edited manually with Seqman (DNASTAR, Inc., Madison, WI, USA). All sequences were aligned using the algorithm Clustal W and a Neighbor Joining Tree (bootstrap 1000×) was constructed using the program MEGA 6.0 [41].

### Data accessibility

All data are provided in the supplementary files of this submission. New MHC allele sequences have been submitted to Genbank under accession numbers MF629153-MF629668 (see Additional file 9: Table S8 for the full list).

## Results

### Population sampling

We used two different sample sets: Sample set 1 represents a general survey, aimed to identify new MHC alleles in wild mice and involved the two subspecies *M. m. domesticus* and *M. m. musculus*, represented by sampling five different regions (depicted as white circles in Fig. 1). For this sample set we sequenced a number of exons from MHC class I (H2-D and H2-K) and MHC class II loci (H2-Aa, H2-Ab, H2-Ea and H2-Eb)(graphic overview and details are provided in Additional file 1: File S1). These sequences were then combined with sequences from the database and served as reference data set for the second part of the study.

Sample set 2 aimed to study the patterns of class II MHC allele distribution in a metapopulation context of *M. m. domesticus*, represented by local groups (demes) of mice from farms in two separate regions (depicted as yellow circles in the extended insets in Fig. 1). Note that the sampling strategy for these samples is different from that for sample set 1. While sample set 1 aimed to obtain unrelated individuals across a region, i.e., only a single individual per deme was sampled, sample set 2 aimed to obtain multiple individuals from given demes within the respective regions. Further, for sample set 2 we restricted the sequence analysis to H2-Aa and H2-Eb exon 2 sequences, since the largest number of previously determined reference alleles from wild mice are available for these loci and since they allow to do direct sequencing of heterozygous individuals, due to the absence of indel polymorphisms.

### Survey of MHC alleles

Based on the sequencing results across populations (sample set 1), we found for all sequenced loci 50–90% more alleles than previously recorded in the NCBI database (Table 1; full sequence lists in Additional file 4: Table S3 and Additional file 5: Table S4). Most alleles were found for class I loci (565 out of 684 for all exons). H2-D showed more alleles for exon 3 than exon 2, H2-K showed the opposite pattern. Both exons code for the domains forming the peptide binding groove. The largest number of alleles (197) were found for exon 4 of H2-K, which codes for a part of the protein which is not directly involved in peptide binding. This exon is usually not surveyed in human studies [14], i.e. knowledge about its polymorphism in other systems is limited. About a third of these alleles appear to be non-functional due to frame shifts or premature stop codons. However, we noticed during the analysis that several individuals showed more than two alleles. We ascribe this to the possibility that the primers we used could also partially amplify paralogs of these genes (the annotated Q, T and M loci - compare depiction in Additional file 1: File S1). These loci vary by copy-number in natural populations [57] and it is therefore unpredictable which variants segregate in the populations. Hence, we can not be certain whether the H2-D and H2-K alleles which we identified here can indeed be ascribed to the respective loci or to their paralogs. On the other hand, since these paralogs appear to be similarly expressed (see expression data in [27]), they might be functionally equivalent. Still, because of the uncertainty of assignment, we did not analyze these alleles in further depth.

**Table 1:** Numbers of MHC H2 alleles found in the general survey (sample set 1)

				fraction of:		
	locus	exon	total	new^a^	silent^b^	non functional^c^
class I	H2-D	2	29	0.66	0.03	0
		3	120	0.90	0.10	0.23
	H2-K	2	183	0.89	0.08	0.08
		3	36	0.64	0	0.11
		4	197	0.84	0.10	0.34
class II	H2-Aa	2	20	0.50	0	0
		3	15	0.53	0.67	0
	H2-Ab	2	41	0.88	0	0.10
		3	28	0.71	0.71	0.21
	H2-Ea	2	7	0.57	0.29	0.14
		3	8	0.63	0	0.25
	H2-Eb	2	55	0.73	0.04	0.13
		3	16	0.81	0.44	0.13

^a^not previously recorded in the NCBI databank

^b^differing only in non-coding sequence positions from other alleles

^c^including a frame shift or stop codon mutation

For the class II loci we had no assignment problem, as there are apparently no paralogs. There are none in the reference sequence and we never found more than two alleles per individual (note that this confirms further that PCR recombination artifacts can be largely excluded - see also Methods). For these loci, we find a larger number of different alleles for exon 2 than for exon 3, with the exception of H2-Ea, which has generally fewer alleles than the three other loci (Table 1).

Since the class II alleles could be unequivocally assigned, we also analyzed their distribution patterns between the populations. Each population shows a subset of already described and new alleles and many are shared between at least one population of each of the subspecies (Table 2 and Additional file 5: Table S4). Interestingly, also alleles that differ only in non-coding positions and even non-functional alleles are partly shared between the subspecies (see annotations in Additional file 5: Table S4).

**Table 2:** Numbers of MHC H2 class II alleles in the different populations of sample set 1

			*M. m. domesticus*			*M. m. musculus*		
Locus	exon	total	IRA^a^ *N* = 12	GER^a^ N = 12	FRA^a^ *N* = 25	CZE^1^ N = 12	KAZ^a^ N = 12	shared^b^
H2-Aa	2	20	4 / 0	6 / 1	14 / 2	7 / 0	6 / 0	7
	3	15	6 / 0	7 / 1	4 / 1	8 / 1	6 / 0	7
H2-Ab	2	41	11 / 6	7 / 6	16/ 7	10 / 4	11 / 5	7
	3	28	9 / 1	11 / 2	15 / 2	19 / 2	10 / 0	17
H2-Ea	2	7	4 / 0	4 / 1	5 / 0	3 / 0	3 / 0	4
	3	8	3 / 0	3 / 1	5 / 1	3 / 0	4 / 1	4
H2-Eb	2	55	16 / 5	12 / 4	30 / 9	17 / 7	14 / 2	15
	3	16	7 / 0	7 / 0	12 / 1	6 / 2	8 / 0	9

^a^first number: total number of alleles found in the population / second number: number of population specific alleles

^b^number of alleles shared between at least one *M. m. domesticus* and at least one *M. m. musculus* population

The largest number of different alleles were found in in the FRA population, which is represented by twice as many individuals as the other populations (Table 2), suggesting that more alleles are found with higher sampling depth, i.e. the discovery of new alleles is not exhausted.

### Deme structure of sample set 2 populations

Sample set 2, collected around Bonn and Espelette (Fig. 1), was used to assess allele distributions among individuals within and between demes. To confirm the deme structure of these locally sampled animals, we typed them for 10 microsatellites and determined mitochondrial D-loop sequences. We find that microsatellite heterozygosities are close to Hardy-Weinberg expectations, with deviations towards higher heterozygosity in two of the demes (Table 3), implying that there is no extreme inbreeding. Still, the average relatedness is relatively high in most demes, supporting the notion of a small effective population size in the demes, up to family level relationships. This leads to high genetic differentiation, even between closely spaced demes (average F_ST_ = 0.4 for Bonn and 0.16 for Espelette - all pairwise F_ST_ comparisons between demes in Additional file 10: Table S9).

**Table 3:** Microsatellite population parameters

region	deme	N	observed heterozygosity	expected heterozygosity	effective number of alleles	average relatedness
Bonn	A	7	0.51	0.52	2.2	0.38
	C	9	0.50	0.50	2.2	0.47
	E	10	0.46	0.45	1.9	0.5
	F	18	0.62	0.55	2.3	0.37
	G	13	0.60	0.56	2.5	0.35
Espelette	JJM02	10	0.71	0.74	3.7	0.16
	JJM09	11	0.86	0.78	4.2	0.15
	MJJ01	16	0.85*	0.72	3.6	0.34
	MJJ06	11	0.72**	0.62	2.7	0.48

significances: * = *p* < 0.05; ** = *p* < 0.01

These findings can be contrasted to the results when one samples only a single animal per deme across the region (same scheme as for sample set 1). In this case one finds a higher number of different microsatellite alleles for a region (e.g. about 10 instead of 2–3) and average observed heterozygosities are always lower than the expected heterozygosities (see Table 1 in [28]). Hence, the comparison between the two sampling schemes (within demes versus across demes) suggests differentiation of demes within a region, typical for metapopulations.

**Fig. 2:**
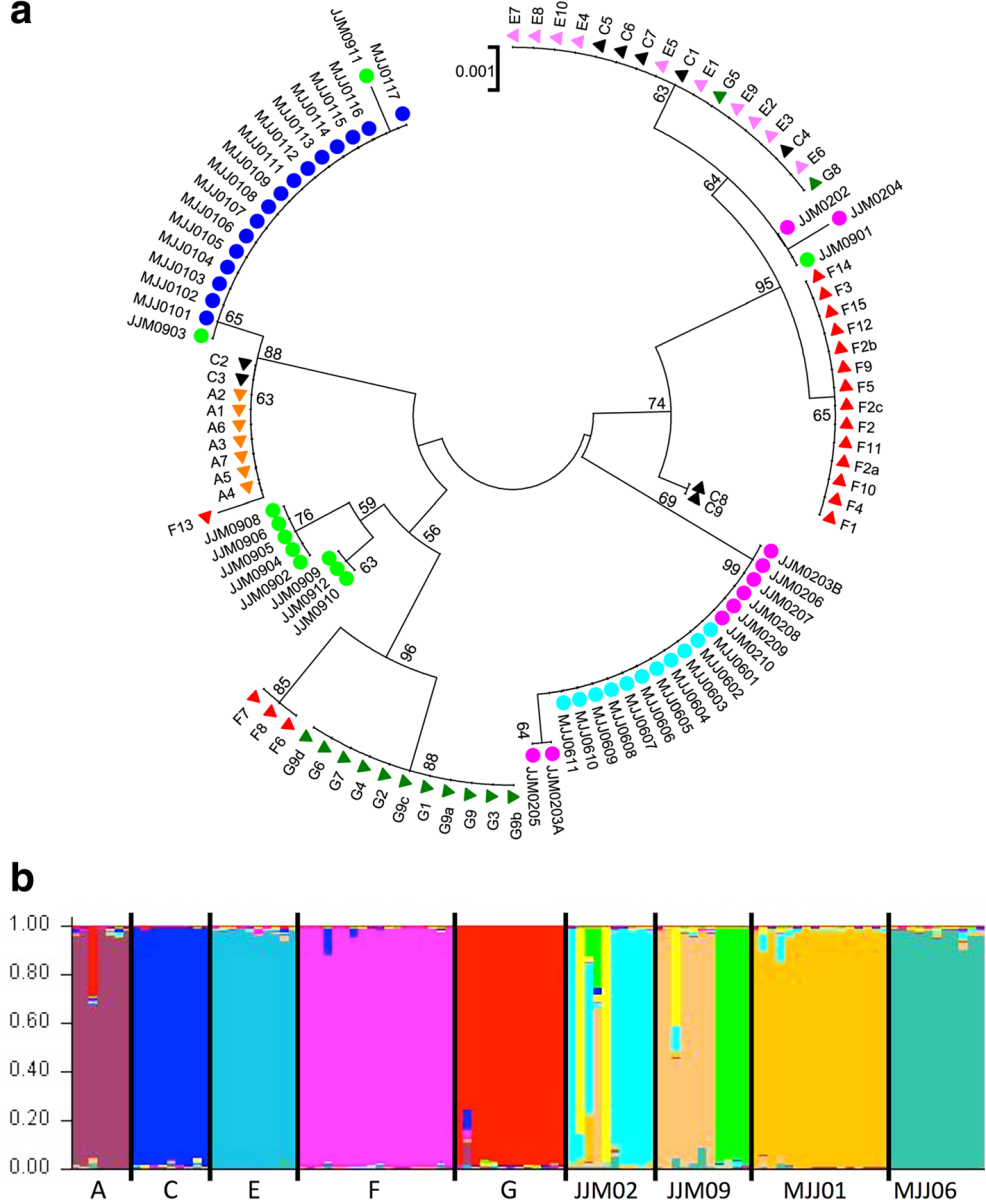
Analysis of demes, based on D-loop sequences (**a**) and microsatellite analysis (**b**). The D-loop sequences show no sharing of haplotypes between France and Germany (circles vs. triangles), but much sharing of haplotypes within demes, confirming a strong matrilineal structure. On the other hand, several haplotypes are also shared between neighboring demes indicating some exchange in the past. The microsatellites were used to assess deme differentiation and degree of more recent exchange based on STRUCTURE analysis. The figure shows the results for K = 11 reflecting the optimal number out of 2–15. The demes JJM02 and JJM09 were split by STRUCTURE into two sub-demes, but with apparent exchange between them. Note that colors are only used for better visualization, they do not correspond between the figure parts

A distinct deme structure is also supported by the patterns found for the D-loop haplotypes, as well as STRUCTURE analysis based on the microsatellite data (Fig. 2). The overall results show that most animals within a deme share a particular D-loop haplotype, indicating a strong matrilineal structure (Fig. 2A). However, there is also some evidence for migration between the demes, such as single animals not fitting into their group or some D-loop haplotypes shared across groups. This is also evident from the STRUCTURE analysis. The pattern confirms a high genetic distinction of the demes, but also that at least a few animals were recently exchanged between some demes (Fig. 2B).

Hence, all data are compatible with a metapopulation structure with extended family groups living at each location and occasional exchange between demes.

### Allele patterns in sample set 2 demes

**Fig. 3:**
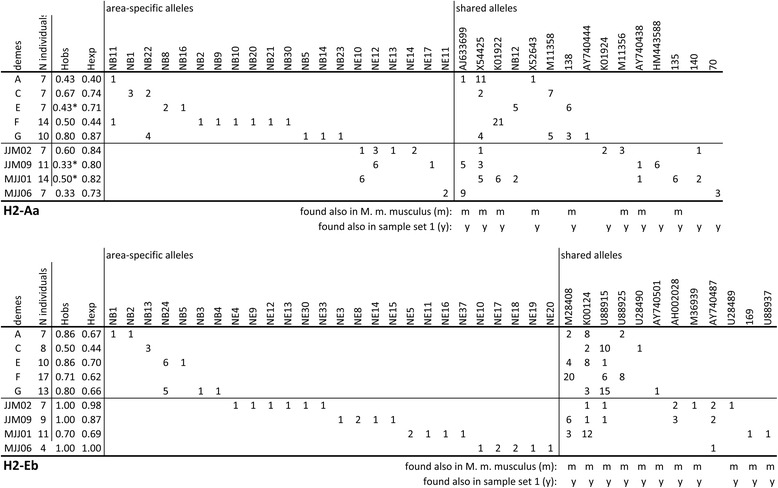
MHCalleles found in the survey of the demes (sample set 2). H2-Aa (top) and H2-Eb (bottom). Deme designations and number of individuals per deme and per locus are listed in the left columns. The next two columns show the observed (Hobs) and expected (Hexp) heterozygosities, significant (*p* < 0.05) deviations from the expected Hardy-Weinberg distribution are marked with a star. Area specific and shared alleles are listed in different columns. The numbers refer to the number of times each allele was found

As described above, for sample set 2, we restricted the analysis to class II exon 2 sequences for H2-Aa and H2-Eb. This was done by direct sequencing of PCR fragments, even from heterozygous individuals. However, in cases of heterozygous individuals, it can be difficult to infer the phase of the respective alleles. Hence, we have applied several precautions to confirm the alleles, including re-sequencing by an Illumina-based approach (see Methods). For allele assignment, we compared the reliably inferred allele sequences with those from our survey, as well as the NCBI sequence database (including the sequences from [22]). This revealed many region-specific or even deme-specific alleles that were not previously identified (Fig. 3, Additional file 6: Table S5 and Additional file 7: Table S6).

**Fig. 4:**
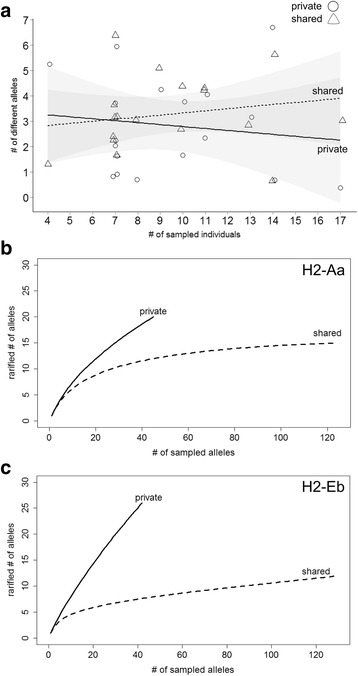
Sampling statistics. (**a**) Correlation between number of sampled individuals per deme and number of alleles found for shared and region-specific alleles. The correlation lines are non-significant (grey area represents the 95% confidence interval). (**b**) and (**c**) Rarefaction curves for the probability of discovery of new alleles with successive sampling. Only sampling of shared alleles comes to a saturation in our data. Note that shared alleles have a higher frequency, i.e. the total numbers are larger than for region-specific alleles, although the number of different alleles is smaller

Different demes harbor between 0 to 7 region-specific alleles and 1 to 6 alleles shared between regions. These numbers do not depend much on sampling depth, since there is no correlation between the number of sampled individuals within a deme and the number of detected alleles, neither for the region-specific nor the shared alleles (overall (R^2^ < 0.001) (Fig. 4a). This implies that within each deme we are approaching saturation of allele detection.

Most demes differ not significantly from Hardy-Weinberg equilibrium for the MHC alleles, and where they do, they show a lowered observed heterozygosity (Fig. 3).

For H2-Aa we find more different alleles in the Bonn region than in Espelette (22 versus 17), while the opposite trend is seen for H2-Eb alleles (13 versus 28), which constitutes a significant difference (two tailed Fisher’s exact test: *p* = 0.04). This confirms the observation of Cizkova et al. [22] that the two loci can evolve rather independently of each other. Note that this difference is mostly driven by the differences in region-specific alleles for these loci (Fig. 3).

There are also other notable differences between region-specific and shared alleles. The majority of shared alleles (24 out of 27) were also found in dataset set 1 and 19 were found in at least one of the *M. m. musculus* populations (see annotations in Fig. 3). In contrast, the majority (41 out of 46) of region-specific alleles were only found in a single deme in their respective region. This suggests that there may be two types of alleles, namely one group that occurs across populations in the whole species (including the different subspecies) and one group that is found more or less exclusively in local demes.

This could be a pure frequency effect, i.e. rare alleles are found by definition only in individual locations. Alternatively, it could correspond to the two classes of alleles that were postulated for humans, namely a core set of ancient alleles and a larger group of region-specific recombinant alleles [14]. If the latter is the case, one would predict that by sampling more demes, one should find increasing numbers of region-specific alleles, while the shared alleles should come to a saturation. Such a trend can indeed be traced in our data. Figure 4b and c show rarefaction plots that indicate that sampling of shared alleles comes to a saturation for both loci, while sampling of region-specific alleles is still far from saturation.

### Sequence differences between alleles

Most of the new alleles in the demes show amino acid differences compared to known alleles, only two new H2-Aa alleles and one new H2-Eb allele show non-coding differences. The amino acid differences are not randomly distributed across the sequenced region. Most occur at known hypervariable sites that constitute the contact sites of the peptide binding pocket of the antigen binding sites (ABS) (Additional file 11: Table S10 and Additional file 12: Table S11). Such biased substitution patterns are typical for MHC alleles and are ascribed to fast positive selection on sites that convey an advantage and negative selection or drift shaping the patterns of the other sites.

However, it is unlikely that such a mutation-selection process could have generated the new alleles within each of the single demes, since this would take an evolutionary time that would go beyond the life-time of these demes. Even when assuming a point mutation rate of 2 × 10^− 8^ and a population size of up to 1000 per deme, one can expect a new mutation to occur within the about 200 bp region surveyed only every 2500 generations. Given that the life time of such a deme may be no more than 1000 years, there would simply not be enough new mutations to reach a mutation/selection equilibrium. Hence, recombination or partial gene conversion between existing alleles, either presently or previously present in the respective deme, is the much more likely explanation for the generation of the region-specific alleles.

We used several analysis algorithms to assess such possible recombination patterns. Given the likely fast allele turnover in these small demes (i.e. loss through drift) and the occasional introgression from neighboring demes (see above), one would not expect that the actual donor sequences for a given recombination event would be found in all cases. Hence, we included all known alleles (obtained from the NCBI database and sample set 1) as possible donors in the analysis of the alleles in each deme. Multiple recombination events could be detected by the four gamete test implemented in DNASp for both loci and most demes (Table 4). Significant sites of recombination in individual demes are detected by GARD analysis (Table 4).

**Table 4:** Estimated recombination events among alleles for each deme

Location	Deme	minimum number of recombination events	significant positions^a^ found by GARD	minimum number of recombination events	significant positions^b^ found by GARD
		H2-Aa		H2-Eb	
Bonn	A	0	–	1	–
	C	2		3	
	E	0	–	3	
	F	3	–	0	
	G	3	–	1	
Espelette	JJM02	8	156**	9	50**
	JJM09	2	156***	4	187***
	MJJ01	3	62*	4	181***
	MJJ06	0	–	9	188***
	all demes	10	–	10	–
	all demes + all known alleles	12	–	13	–

^a^position numbering refers to the sequences in Additional file 7: Table S6

^a^position numbering refers to the sequences in Additional file 8: Table S7

*p < 0.05; **p < 0.01; ***p < 0.001

Using the program GENECONV, we found also support for a number of partial gene conversion events within the demes (Table 5). Three significant events were found for H2-Aa and one for H2-Eb, but only in the Espelette region.

**Table 5:** Partial gene conversion events among alleles within demes

locus	deme	kind^a^	allele 1	allele 2	simulated *p*-value	Begin	End	Length
H2-Aa	JJM02	GI	NE12	M11356	0.0016	57	183	127
	JJM02	GI	NE13	M11356	0.0431	112	183	72
	JJM09	GI	NE12	AY740438	0.0442	28	183	156
H2-Eb	MJJ01	GO	NE16		0.0422	28	42	15

^a^GI: global inner fragment = runs of matching sites; GO: global outer fragment = runs of matching sites unique in that group

## Discussion

Our study confirms the original findings of Duncan et al. [16], namely a high number of MHC alleles in wild caught mice, and that class I alleles are more polymorphic than class II alleles. However, class I allelic diversity is also influenced by paralogs with variable copy-numbers, which were not known at that time. Hence, the serological analysis done at that time could have picked up variants from expressed paralogs, i.e. the true degree of heterozygosity at these loci remains unclear. Given these complications, we have not explored them further in this study. Duncan et al. [16] also found a difference in allelic diversity between class II A and E loci, with relatively fewer alleles for locus E. We can confirm this for the H2-Ea locus, while we find that H2-Eb is on average highly polymorphic in our survey (compare Table 2).

We find many alleles that are shared across sub-species, which is a pattern generally known for MHC alleles [3, 18]. These have usually been ascribed to be the result of balancing selection and/or incomplete lineage sorting after the splitting of the species. However, for species that have remained at least partially inter-fertile, there is increasing evidence that introgression must play a role as well [58–61]. In fact, geographical long-range transfer of haplotypes and adaptive introgression across sub-species was also found for other parts of the genome in mice [31]. In humans it is also thought that some alleles of the immune system are derived from introgression events among archaic lineages [58].

The mitochondrial and microsatellite analysis of the deme structure confirms the notion that these are composed of extended family groups, with average relatedness values up to 0.5, but still largely in Hardy-Weinberg equilibrium. Intriguingly, and contrary to the expectations by Duncan et al. [16] that this deme structure should reduce allelic MHC diversity due to inbreeding and drift, we actually find more different region-specific alleles than shared alleles among these demes, although most of them are at a low frequency. Previous studies may have largely missed these low frequency alleles, because of less deep sampling, or because of the use of filtering steps that would have removed them as possible PCR artifacts. We applied such filters also for the sample set 1 study. In particular, we required at least two independent PCRs with the same sequence from at least two animals, which biases against rare alleles, since more abundant alleles are more likely to be found repeatedly. No such filter was used for sample set 2 (i.e. the alleles in the demes), since these were detected through direct sequencing and confirmed by Illumina sequencing of individuals, which allows the reliable identification of alleles that occur only in a single individual.

There is little sharing of region-specific alleles (5 out of 46) even between neighboring demes. In contrast, the shared alleles are not only shared more often within each region (16 out of 27), but most of them are also found in the populations of set 1 (23 out of 27) and across sub-species (19 out of 27). This is not simply an effect of relative frequencies. Although region-specific alleles tend to have a low frequency in demes, their probability of discovery does not correlate with sample size in the demes (Fig. 4a), i.e. we expect that the sampling is reasonably representative for each deme. Further, the rarefaction analysis suggests that sampling saturation is not reached for region-specific alleles (Fig. 4b, c), i.e. they can be predicted to occur in increasingly large numbers when more demes are sampled. Hence, considering the multitude of different demes in which mice can be found across their whole distribution range, one may conclude that region-specific alleles should by far outnumber shared alleles.

This raises the question how the region-specific alleles arise in the demes. The analysis of their sequence differences suggests that they are mostly not created by new point mutations, but by recombination and/or partial gene conversion from existing alleles. These mechanisms were first described in detail for human class I loci [3], whereby the donors for the recombination or partial gene conversion were suggested to come mostly from intragenomic paralogs. In the mouse, there are no paralogs for the class II H2-Aa and H2-Eb loci that could serve as donors. Although a duplicate exists for H2-Eb (annotated as Eb1 and Eb2), this is molecularly too diverged to allow recombination to happen. Hence, the donors for recombination would have to be other alleles segregating within the demes, or coming in via occasional migration. We find indeed evidence for such recombination and partial gene conversion events within demes when taking all known alleles of the orthologs into account (see Tables 4 and 5). A further possibility are short tracks of gene conversion even between distant paralogs. The Aß^bm12^ allele in the C57BL/6 laboratory strain has been suggested to have been created by a micro gene conversion event between H2-A and H2-E [62], i.e. this appears to work even between very diverged copies.

Recombination and partial gene conversion among MHC alleles has been documented in many other cases (e.g. [14, 22, 63–69]. Spurgin et al. [69] showed rather directly that allelic diversity is re-created through partial gene conversion events after bottlenecks in birds. Bergström et al. [66] and Spurgin et al. [69] estimated that the generation of new alleles through recombination mechanisms is at least one order of magnitude faster than through point mutations. Direct measurements of gene conversion rates between HLA genes in sperm have yielded an estimate two orders of magnitude higher than the point mutation rate in mice [64] and more than three orders of magnitude higher in humans [65]. This fits well with our observation that point mutations are rare in comparison to recombination patterns.

Exceptionally deep sampling of MHC diversity has been done in humans, since this is essential for matching donors and recipients for hematopoietic stem cell transplantations. Klitz et al. [12] have proposed that this diversity is mostly shaped by recombination mechanisms that create continuously rare variants and generate in this way a reservoir of new alleles that are likely to be pre-adapted for presentation of new pathogen-derived peptides. They provide some basic population genetic calculations implying that the human population as a whole might harbor millions of alleles at present. But this calculation depends on many assumptions about effective population size, equilibrium state, mutation rate, deme sizes and migration rates. Hence, while the same parameters might apply to mice, we refrain from such calculations since they are too speculative. However, it would not seem unlikely that mice as a whole might also harbor similar numbers of MHC alleles as humans.

A recent detailed analysis of human class I alleles in databases has also suggested that millions of alleles appear to exist [14]. This was estimated on the basis of the discovery rate of new alleles in tested cohorts, which was found to lie around 2 per 10,000 individuals tested. This is relatively low compared to our discovery rate of around 2.7 new alleles per 10 individuals (across all demes and loci), i.e. the mouse sampling must indeed be far from saturation.

In humans there is also evidence for a set of common alleles occurring at elevated frequencies and across populations [70]. This is comparable to the set of shared alleles that we find in mice, most of which occur also across subspecies. This suggests that they have a specific adaptive value, which maintains them or allows them to introgress between the diverse lineages. On the other hand, the rare alleles occur only locally, both in humans [14, 70] and mice (this study).

## Conclusion

Although recombination mechanisms have long been known to contribute to allelic diversity at MHC loci, the current results suggest that it generates a large number of new alleles in demes, with much higher efficiency than point mutations would do this. However, most may also get quickly lost, due to small effective population sizes in the demes. Still, they constitute a reservoir of possible resistance alleles when new parasites come along. This leads to a new paradigm on how we should view the generation and maintenance of the diversity at MHC loci [12]. Given that these patterns match between mice and humans [12, 14], it would seem likely that they are generally typical for animals harboring the MHC adaptive immune system.

## Additional files

Additional file 1: **File S1.** Experimental approach for sample set 1 survey (PPT 3321 kb)

Additional file 2: **Table S1.** PCR and sequencing primers (XLSX 17 kb)

Additional file 3: **Table S2.** 454 read statistics (XLSX 38 kb)

Additional file 4: **Table S3.** List of all validated class I alleles found in the 454 approach (XLSX 80 kb)

Additional file 5: **Table S4.** List of all validated class II alleles found in the 454 approach (XLSX 58 kb)

Additional file 6: **Table S5.** H2-Aa alleles in demes (XLSX 24 kb)

Additional file 7: **Table S6.** H2-Eb alleles in demes (XLSX 24 kb)

Additional file 8: **Table S7.** Microsatellite alleles in demes (XLSX 20 kb)

Additional file 9: **Table S8.** Genbank accession numbers for new alleles (XLSX 46 kb)

Additional file 10: **Table S9.** Pairwise distances between demes based on microsatellites (FST) (XLSX 37 kb)

Additional file 11: **Table S10.** Alignments of H2-Aa and H2-Eb alleles (XLSX 47 kb)

Additional file 12: **Table S11.** dN/dS and K2P nucleotide distance/Poisson corrected amino acid distances (XLSX 52 kb)
