# Supplementary material for: Meta-populational demes constitute a reservoir for large MHC allele diversity in wild house mice (Mus musculus)
Source: Front Zool. 2018 Apr 20;15:15. doi: 10.1186/s12983-018-0266-9 (PMC5910556; doi:10.1186/s12983-018-0266-9)
Supplement: Supplementary file 1 — File S1. Experimental approach for sample set 1 survey (PPT 3321 kb) [file 12983_2018_266_MOESM1_ESM.ppt]

## Slide 1
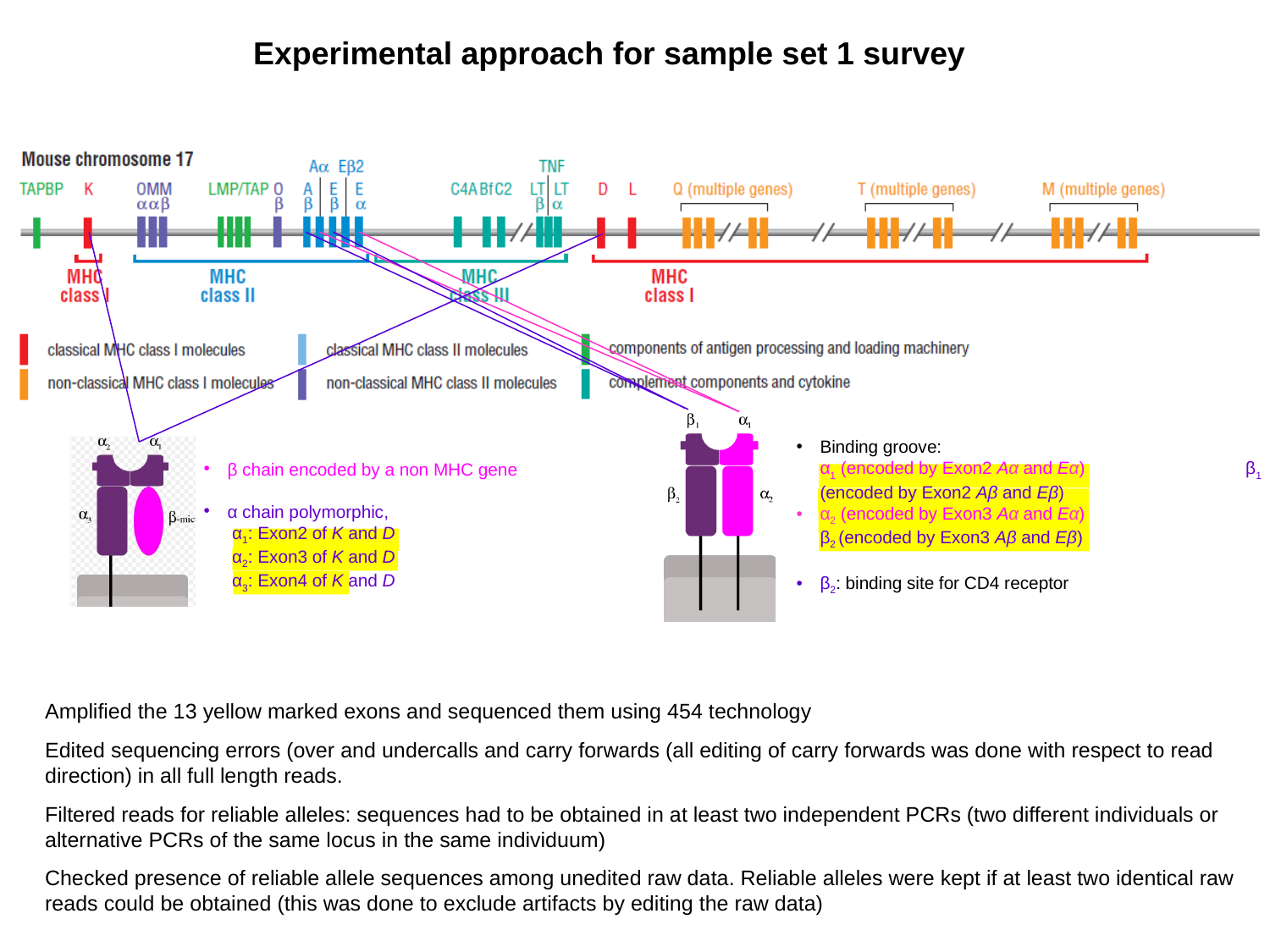

Experimental approach for sample set 1 survey
Binding groove:
	α1 (encoded by Exon2 Aα and Eα) β1 (encoded by Exon2 Aβ and Eβ)
α2 (encoded by Exon3 Aα and Eα)
	β2 (encoded by Exon3 Aβ and Eβ)
β2: binding site for CD4 receptor
β chain encoded by a non MHC gene
α chain polymorphic,
	 α1: Exon2 of K and D
	 α2: Exon3 of K and D
	 α3: Exon4 of K and D
Amplified the 13 yellow marked exons and sequenced them using 454 technology
Edited sequencing errors (over and undercalls and carry forwards (all editing of carry forwards was done with respect to read direction) in all full length reads.
Filtered reads for reliable alleles: sequences had to be obtained in at least two independent PCRs (two different individuals or alternative PCRs of the same locus in the same individuum)
Checked presence of reliable allele sequences among unedited raw data. Reliable alleles were kept if at least two identical raw reads could be obtained (this was done to exclude artifacts by editing the raw data)

## Slide 2
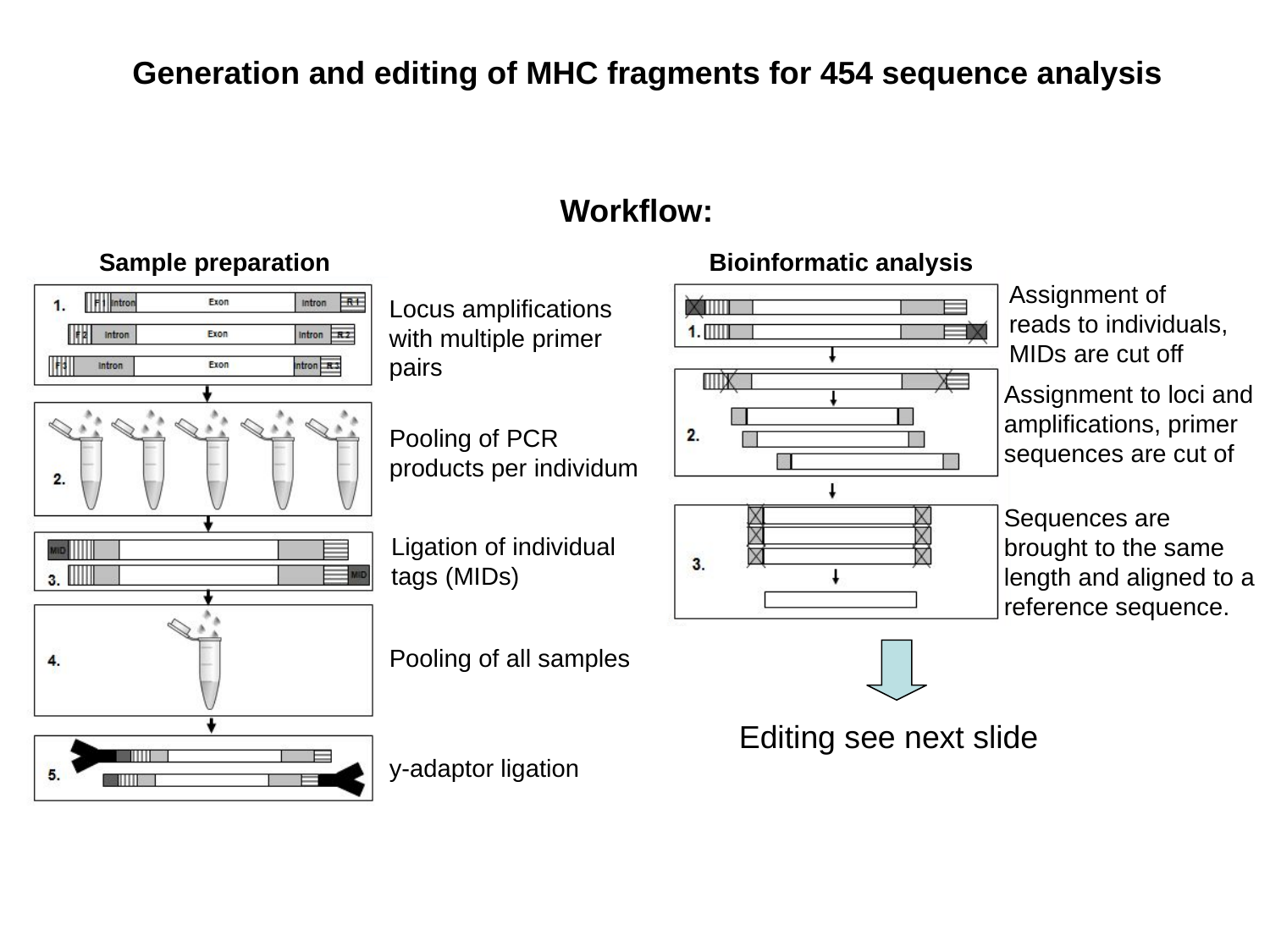

Generation and editing of MHC fragments for 454 sequence analysis
Workflow:
Sample preparation
Bioinformatic analysis
Assignment of reads to individuals, MIDs are cut off
Locus amplifications with multiple primer pairs
Assignment to loci and amplifications, primer sequences are cut of
Pooling of PCR products per individum
Sequences are brought to the same length and aligned to a reference sequence.
Ligation of individual tags (MIDs)
Pooling of all samples
Editing see next slide
y-adaptor ligation

## Slide 3
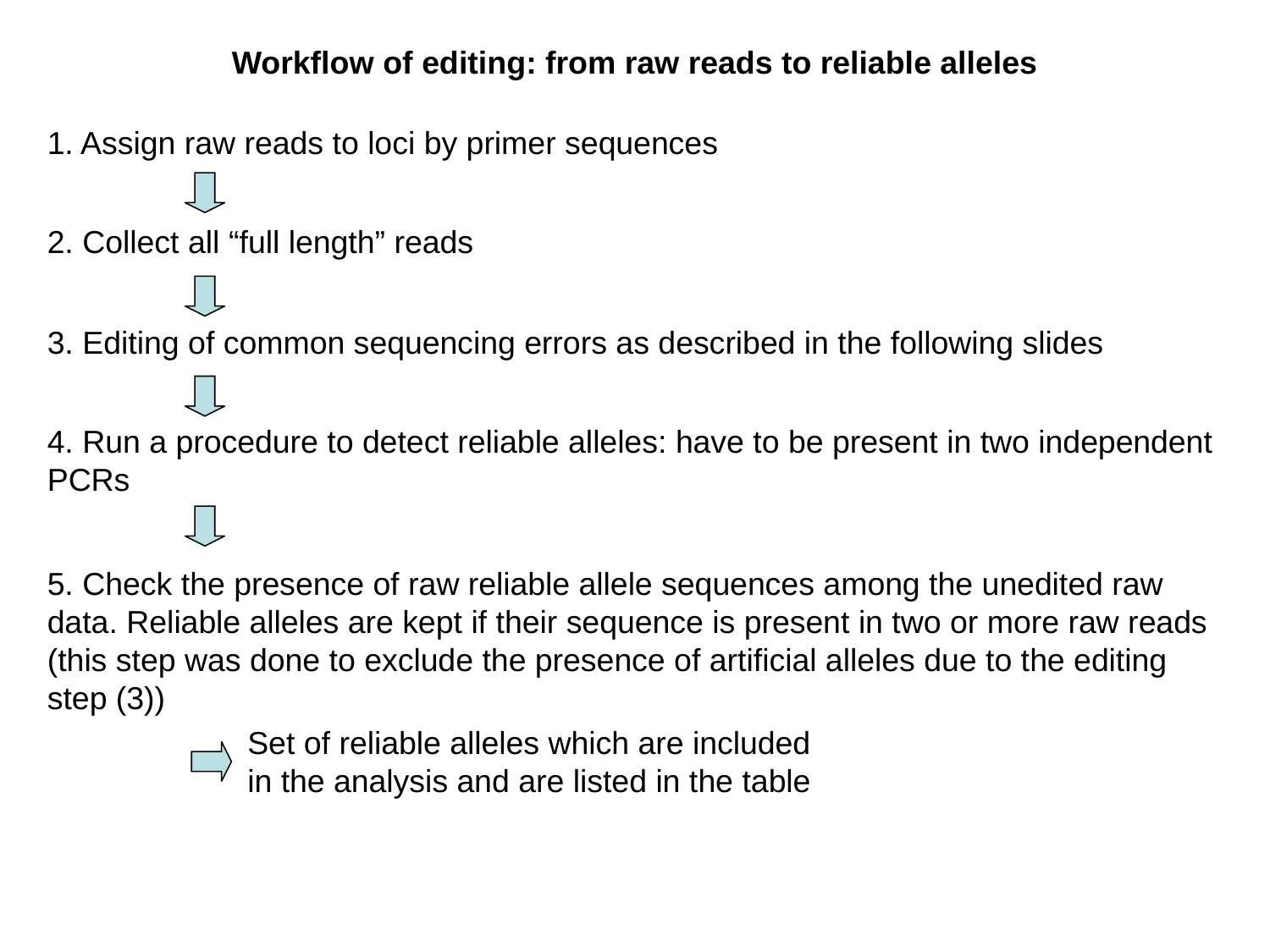

Workflow of editing: from raw reads to reliable alleles
1. Assign raw reads to loci by primer sequences
2. Collect all “full length” reads
3. Editing of common sequencing errors as described in the following slides
4. Run a procedure to detect reliable alleles: have to be present in two independent PCRs
5. Check the presence of raw reliable allele sequences among the unedited raw data. Reliable alleles are kept if their sequence is present in two or more raw reads (this step was done to exclude the presence of artificial alleles due to the editing step (3))
Set of reliable alleles which are included in the analysis and are listed in the table

## Slide 4
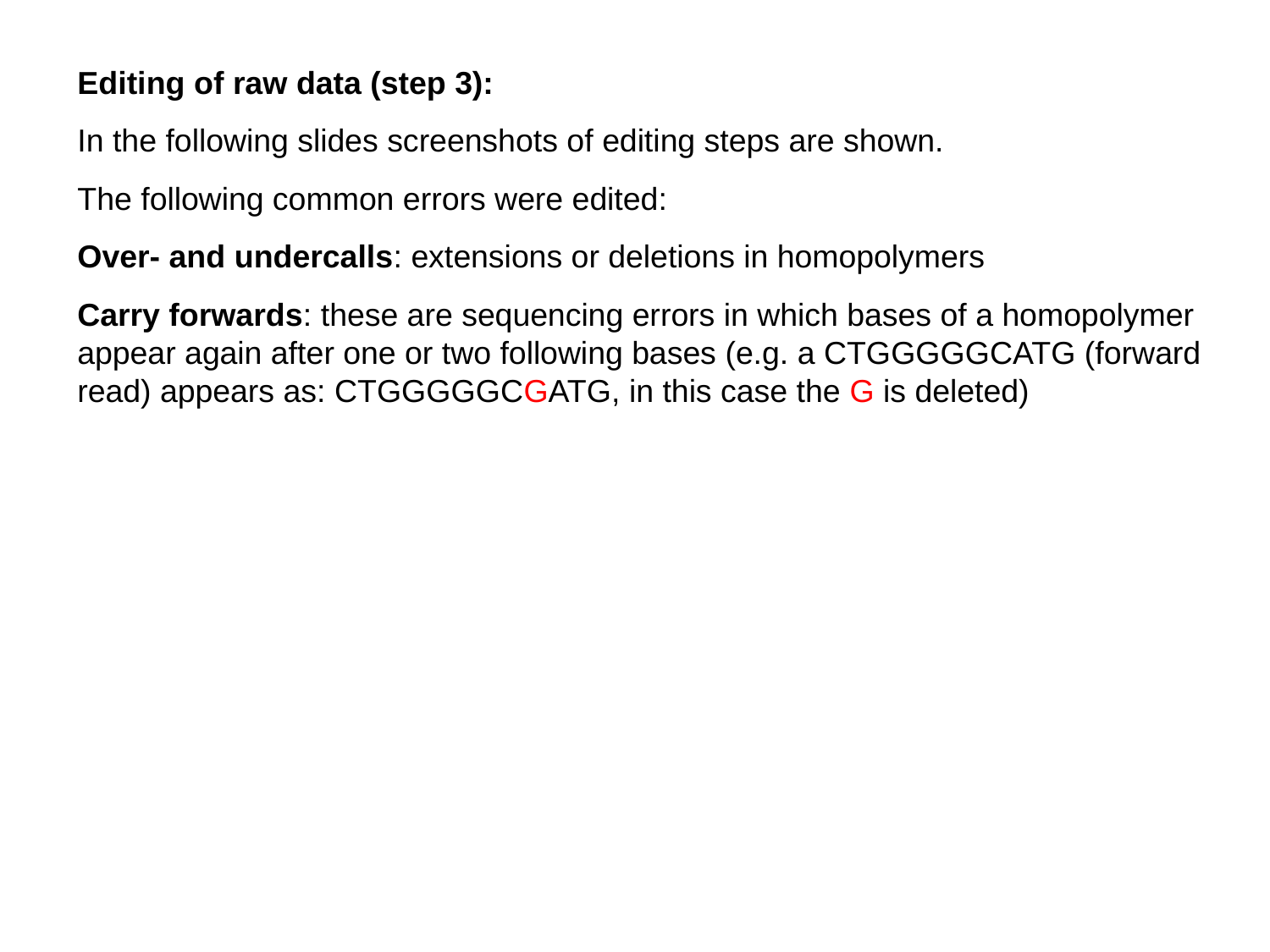

Editing of raw data (step 3):
In the following slides screenshots of editing steps are shown.
The following common errors were edited:
Over- and undercalls: extensions or deletions in homopolymers
Carry forwards: these are sequencing errors in which bases of a homopolymer appear again after one or two following bases (e.g. a CTGGGGGCATG (forward read) appears as: CTGGGGGCGATG, in this case the G is deleted)

## Slide 5
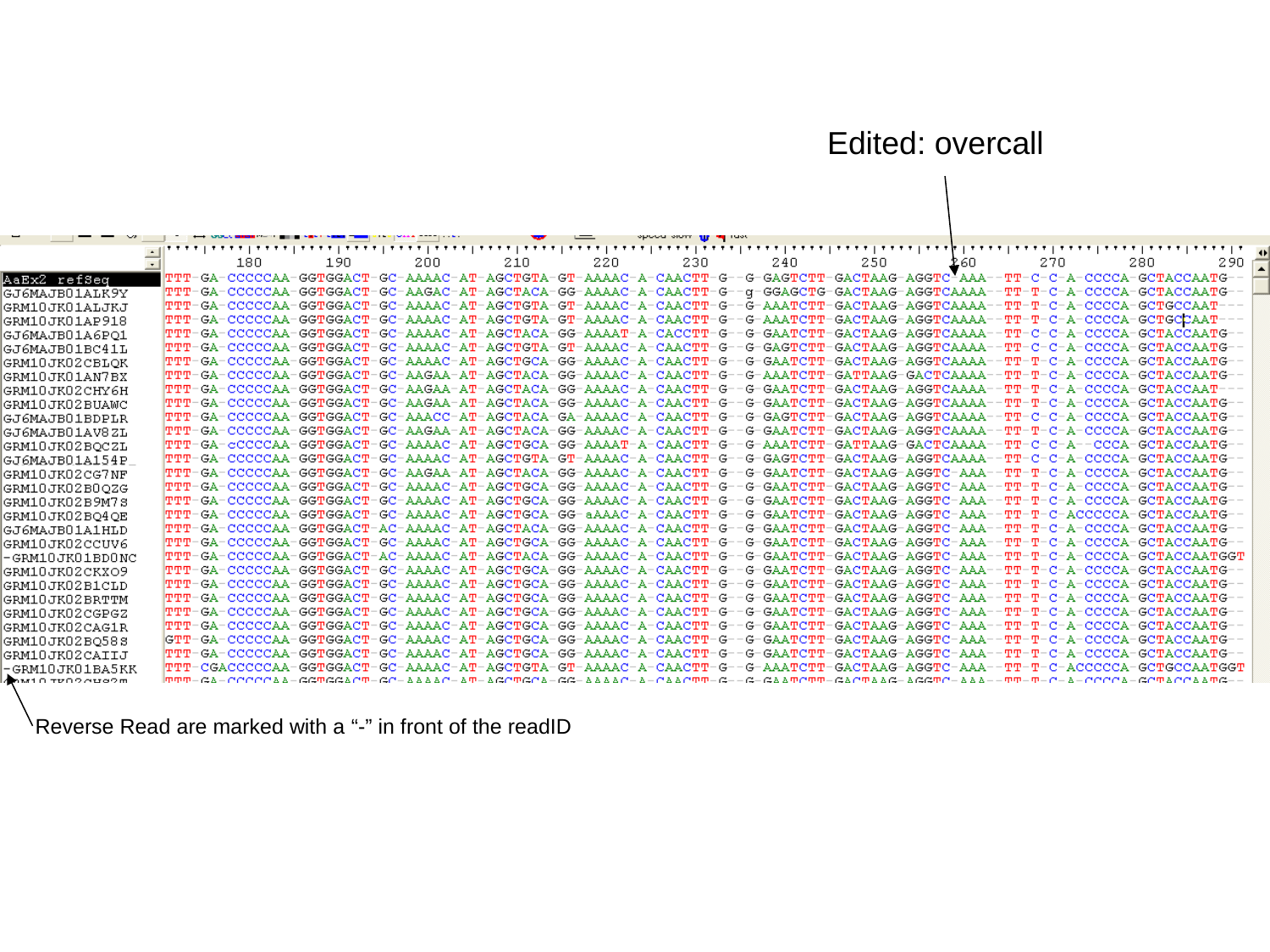

Edited: overcall
Reverse Read are marked with a “-” in front of the readID

## Slide 6
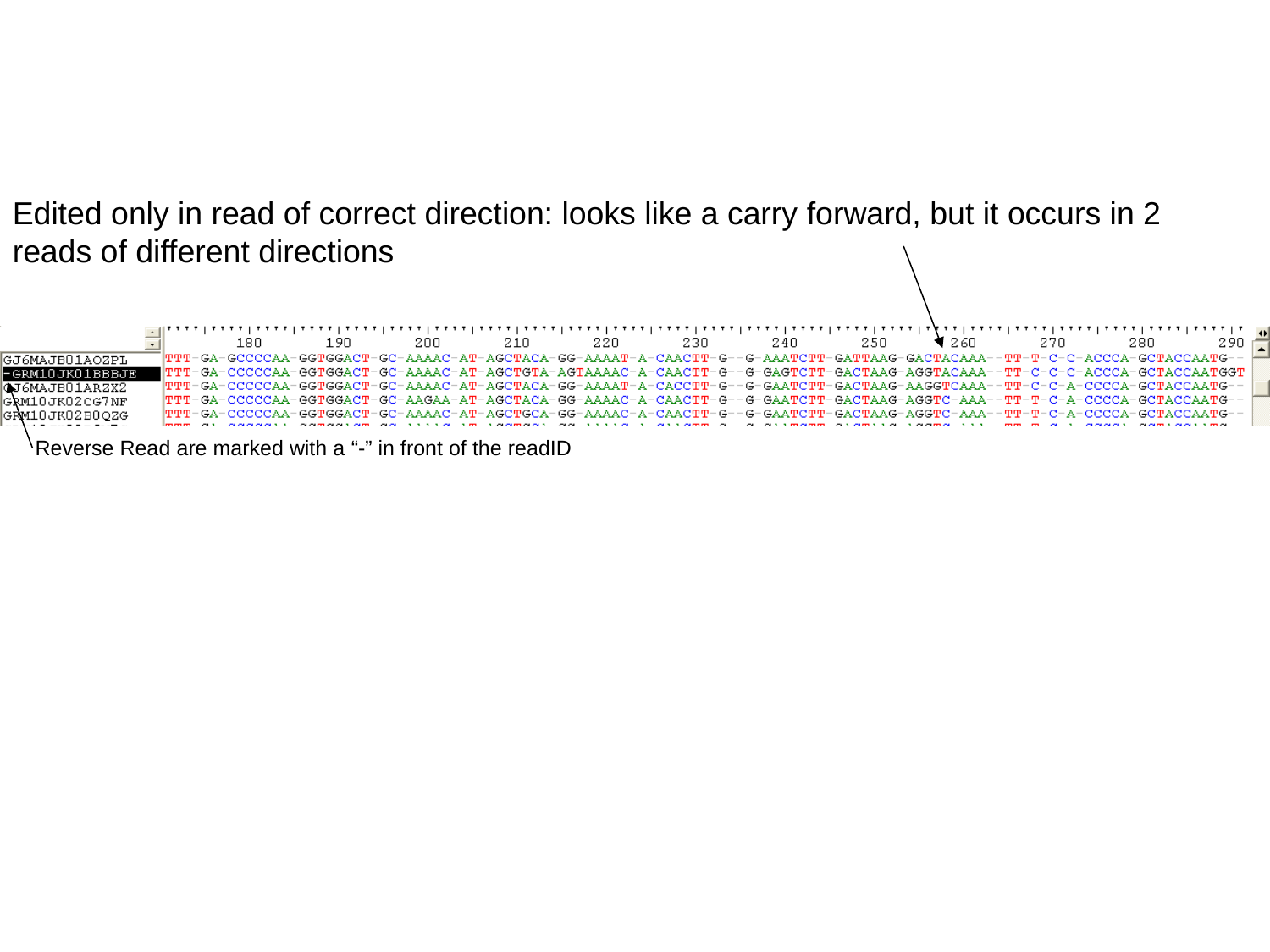

Edited only in read of correct direction: looks like a carry forward, but it occurs in 2 reads of different directions
Reverse Read are marked with a “-” in front of the readID

## Slide 7
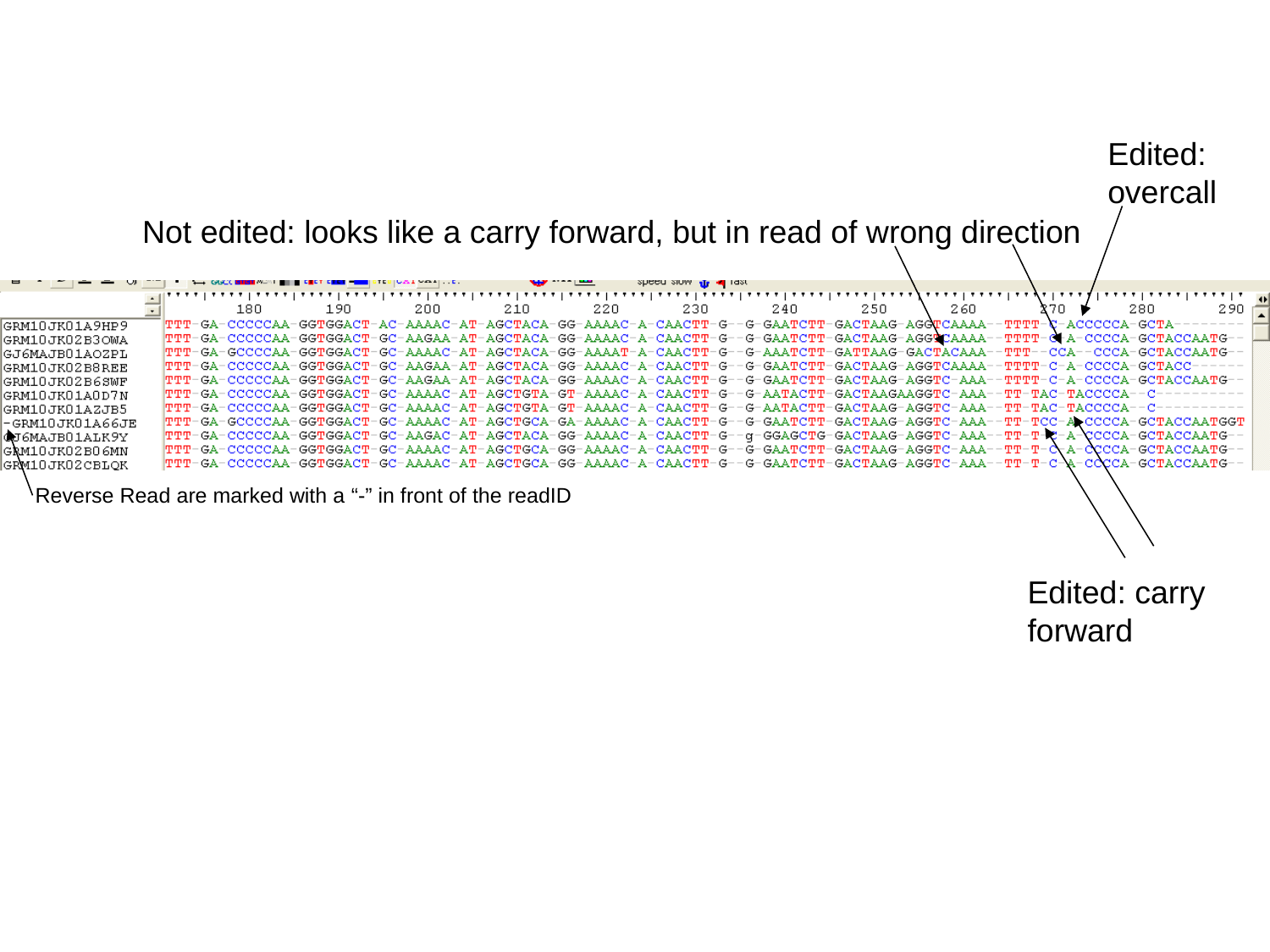

Edited: overcall
Not edited: looks like a carry forward, but in read of wrong direction
Reverse Read are marked with a “-” in front of the readID
Edited: carry forward

## Slide 8
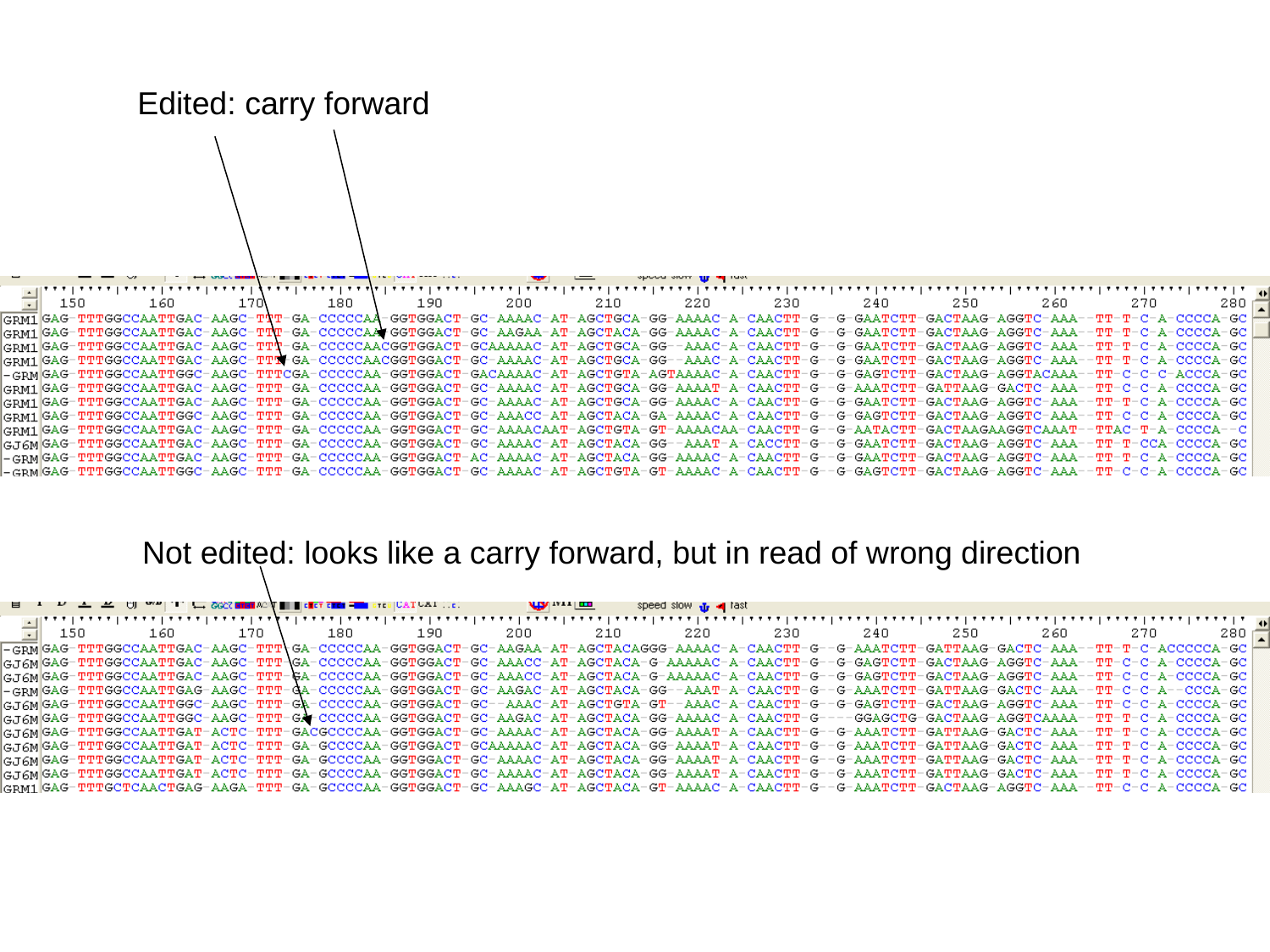

Edited: carry forward
Not edited: looks like a carry forward, but in read of wrong direction

## Slide 9
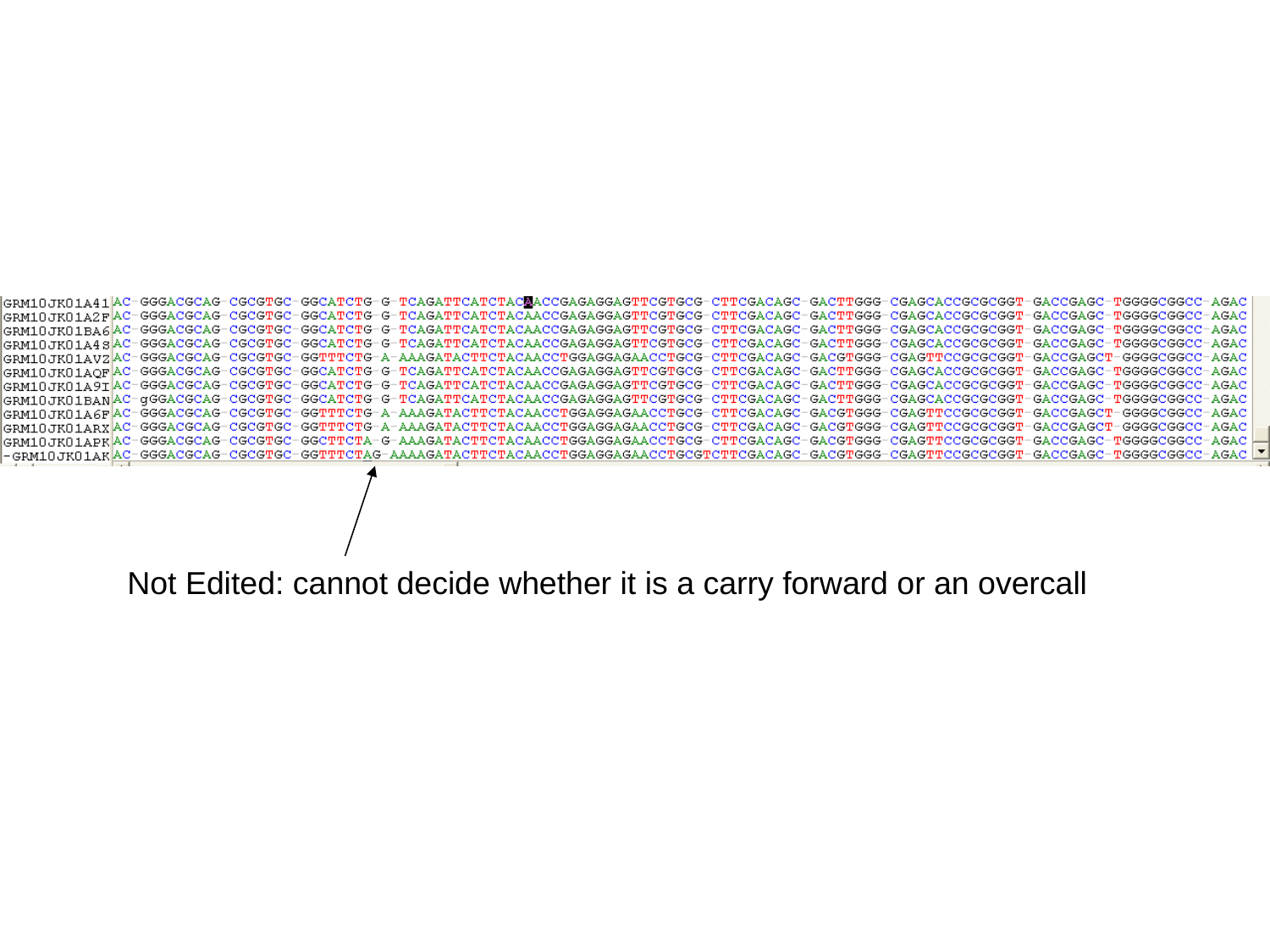

Not Edited: cannot decide whether it is a carry forward or an overcall
